# The Role of the UPR Pathway in the Pathophysiology and Treatment of Bipolar Disorder

**DOI:** 10.3389/fncel.2021.735622

**Published:** 2021-08-31

**Authors:** Mahmoud Suliman, Michael W. Schmidtke, Miriam L. Greenberg

**Affiliations:** Department of Biological Sciences, Wayne State University, Detroit, MI, United States

**Keywords:** bipolar disorder, endoplasmic reticulum stress, mood disorder, unfolded protein response, valproate, lithium

## Abstract

Bipolar disorder (BD) is a mood disorder that affects millions worldwide and is associated with severe mood swings between mania and depression. The mood stabilizers valproate (VPA) and lithium (Li) are among the main drugs that are used to treat BD patients. However, these drugs are not effective for all patients and cause serious side effects. Therefore, better drugs are needed to treat BD patients. The main barrier to developing new drugs is the lack of knowledge about the therapeutic mechanism of currently available drugs. Several hypotheses have been proposed for the mechanism of action of mood stabilizers. However, it is still not known how they act to alleviate both mania and depression. The pathology of BD is characterized by mitochondrial dysfunction, oxidative stress, and abnormalities in calcium signaling. A deficiency in the unfolded protein response (UPR) pathway may be a shared mechanism that leads to these cellular dysfunctions. This is supported by reported abnormalities in the UPR pathway in lymphoblasts from BD patients. Additionally, studies have demonstrated that mood stabilizers alter the expression of several UPR target genes in mouse and human neuronal cells. In this review, we outline a new perspective wherein mood stabilizers exert their therapeutic mechanism by activating the UPR. Furthermore, we discuss UPR abnormalities in BD patients and suggest future research directions to resolve discrepancies in the literature.

## Introduction

Bipolar disorder (BD) is a mood disorder that is characterized by moods alternating between mania and depression (Baldessarini et al., 2020). BD affects 2% of the population and is associated with a high rate of suicide (Gonda et al., 2012; Baldessarini et al., 2020). While there is no single biological marker correlated with BD, there is strong evidence of heritability, and multiple genes have been found to be linked to increased risk for the disease (Grande et al., 2016; Stahl et al., 2019). Environmental factors also play a role in the onset of the disease (Vieta et al., 2018). Lithium (Li) and valproate (VPA) are among the primary drugs used to treat BD (Geddes and Miklowitz, 2013). Yet, these drugs are not effective for all patients and can cause serious side effects, including hepatoxicity, renal toxicity, teratogenicity, cognitive impairment, hair loss, and weight gain (Dreifuss et al., 1987; Pijl and Meinders, 1996; Mercke et al., 2000; Yonkers et al., 2004; Gitlin, 2016). Therefore, better drugs are needed to treat BD patients. However, the mechanism of action of BD drugs is not known, which hinders the development of effective drugs with minimal side effects.

Many studies have aimed to characterize the cellular effects of BD drugs in order to improve our understanding of their therapeutic mechanism, and numerous cellular targets have been proposed, including the neurotransmitter and neuromodulator systems, neuronal plasticity pathways, and *myo*-inositol metabolism (Berridge, 2014). Studies have also suggested that the unfolded protein response (UPR) pathway may be part of the pathophysiology of BD and that mood stabilizers could exert their therapeutic mechanism by activating the UPR (Wang et al., 1999; Bown et al., 2000; Chen et al., 2000; Kakiuchi et al., 2003; Shao et al., 2006; Kim et al., 2009; Pfaffenseller et al., 2014; Bengesser et al., 2016). The pathophysiology of BD is associated with mitochondrial dysfunction, oxidative stress, and abnormalities in calcium signaling, and the UPR plays a role in all of these pathways (Berk et al., 2011; Callaly et al., 2015; Vincenz-Donnelly and Hipp, 2017; Harrison et al., 2019). Therefore, deficient UPR activation could be a common mechanism underlying the array of cellular dysfunctions associated with BD. In support of this, several studies have reported a deficiency in UPR activation in lymphoblasts from BD patients (Table 1), as well as altered expression of UPR target genes following treatment with BD drugs (Table 2).

**TABLE 1:** Findings relevant to the role of UPR function in bipolar disorder.

Study findings	Sample type	Sample	References
• XBP1 -116C/G SNP is associated with BD.	Lymphocytes from peripheral blood	• 451 healthy • 197 BD	Kakiuchi et al., 2003
• Lower basal transcription of XBP1 and GRP78 in BD	Lymphoblastoid cells from peripheral blood	• One pair of healthy twins • Two pairs of twins with BD	Kakiuchi et al., 2003
• Attenuated XBP1 and CHOP mRNA induction in BD after TG and Tun treatment	B-lymphoblast cells	• 10 healthy • 20 BD	So et al., 2007
• XBP1 and GRP94 mRNA induction was lower in BD following TG treatment.	Lymphocyte cells	• 59 healthy • 59 BD	Hayashi et al., 2009
• GRP78 basal mRNA levels are higher in BD. • Total and non-spliced XBP1 are lower in BD.	Peripheral blood mononuclear cells	• 54 healthy • 81 BD	Bengesser et al., 2018

**TABLE 2:** Mood stabilizers alter the expression of UPR target genes.

Effect of treatment relative to control	Sample	References
• VPA increases GRP78 protein levels.	Rat cerebral cortex and rat C6 glioma	Wang et al., 1999
• VPA increases GRP78 protein levels.	HEK293	Shi et al., 2007
• VPA increases GRP78 and calreticulin mRNA and protein levels. • VPA increases GRP94 mRNA levels.	Rat C6 glioma	Bown et al., 2000
• VPA increases GRP78, GRP94, and calreticulin protein levels.	Rat brain	Chen et al., 2000
• VPA and Li increase GRP78, GRP94, and calreticulin protein levels. • Li increases GRP78, GRP94, and calreticulin mRNA levels.	Primary cultured rat cerebral cortical cells	Shao et al., 2006
• VPA increases ATF6 mRNA levels	SH-SY5Y and human lymphoblastoid	Kakiuchi et al., 2003
• VPA increases WFS1 mRNA and protein levels.	Neuro-2a	Kakiuchi et al., 2009
• VPA increases mRNA levels of the ER chaperone genes EUG1, • JEM1, KAR2, LHS1, SEC63, and PDI1.	*Saccharomyces cerevisiae*	Jadhav et al., 2016

In this review, we highlight the existing data regarding UPR activation by mood stabilizers, discuss UPR abnormalities in BD patients, and suggest future research directions to clarify conflicting findings obtained from different studies. We analyze several mechanisms that could explain how mood stabilizers activate the UPR, focusing on a novel hypothesis wherein *myo*-inositol depletion serves as the mechanistic trigger for UPR activation.

## The UPR Pathway

The UPR is a stress response signaling pathway that is conserved from yeast to mammals (Foti et al., 1999). The UPR has a dual function, as it promotes homeostasis and cell survival under mild ER stress but can lead to apoptosis and cell death under intense, persistent stress (Chan et al., 2015; Hiramatsu et al., 2015). Various stressors activate the UPR, such as the accumulation of unfolded proteins in the ER lumen, lipid disequilibrium, calcium imbalance, nutrient limitation, and oxidative stress (Kaufman et al., 2002; Yan et al., 2008; Gardner and Walter, 2011; Hou et al., 2014; Krebs et al., 2015). The UPR activates downstream signaling cascades that induce genes functioning in protein folding, degradation, and translation arrest to reduce the protein load in the ER (Perri et al., 2015).

**FIGURE 1:**
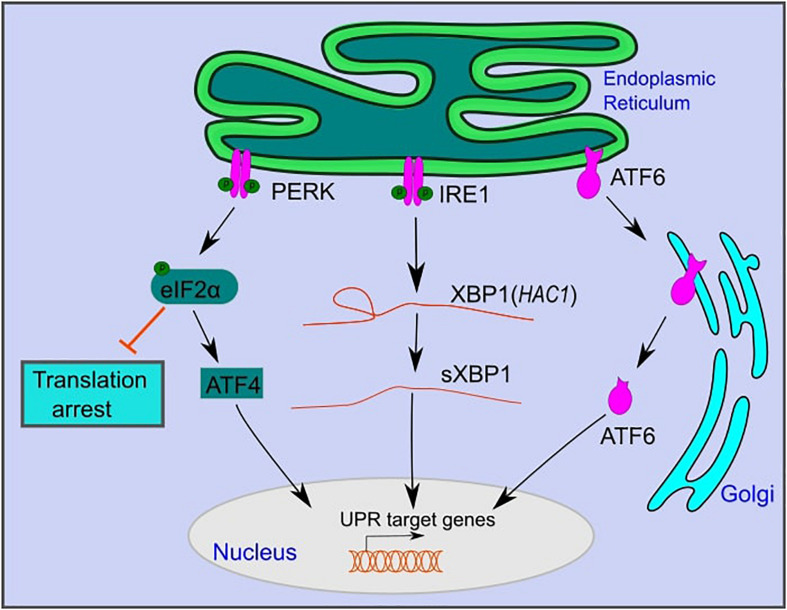
The mammalian unfolded protein response pathway. The UPR is activated upon ER stress caused by the accumulation of unfolded proteins in the lumen of the ER. UPR activation is mediated by three branches: PKR-like endoplasmic reticulum kinase (PERK), inositol-requiring kinase 1 (IRE1), and activating transcription factor 6 (ATF6). PERK and IRE1 are activated by autophosphorylation. Active PERK phosphorylates eIF2α, which inhibits overall protein translation, while selectively promoting the translation of activating transcription factor 4 (ATF4). Active IRE1 splices an intronic region from XBP1 mRNA (*HAC1* in yeast) to form sXBP1, which is translated into an active transcription factor. ATF6 is translocated to the Golgi, where it is cleaved and further translocated to the nucleus. ATF6, sXBP1, and ATF4 activate downstream signaling cascades that increase the expression of genes that function in restoring ER homeostasis or inducing cell death under persistent ER stress.

In yeast, UPR activation is mediated by IRE1, a type-1 transmembrane kinase, and endoribonuclease (Figure 1; Yamamoto et al., 2004; Korennykh et al., 2009). Activated IRE1 excises a 252 bp intronic region from *HAC1* mRNA, which is translated to the active transcription factor Hac1 that translocates to the nucleus and activates UPR target genes (Kawahara et al., 1997; Foti et al., 1999). These genes, which contain UPR response elements (UPRE) in their promotors, include ER chaperones such as *KAR2* (Figure 1; Kawahara et al., 1997; Foti et al., 1999). Chaperones assist in protein folding and maturation in the ER (Braakman and Hebert, 2013).

IRE1 is the only UPR branch that is conserved from yeast to mammals (Yamamoto et al., 2004; Korennykh et al., 2009). Similar to yeast, upon ER stress, mammalian IRE1 is activated by autophosphorylation (Yamamoto et al., 2004; Korennykh et al., 2009). It catalyzes the splicing of a 26 bp intronic region of XBP1 mRNA to form spliced XBP1 (sXBP1), which is translated to an active transcription factor (Calfon et al., 2002; Hiramatsu et al., 2011). sXBP1 translocates to the nucleus and activates UPR target genes that contain conserved ER stress response elements (ERSE) in their promoters. These are similar to UPREs in yeast and include genes that function in protein folding, lipid metabolism, and ER-associated degradation (Yamamoto et al., 2004; Korennykh et al., 2009; Piperi et al., 2016).

In addition to IRE1, mammals have two additional UPR branches: activating transcription factor 6 (ATF6) and protein kinase RNA (PKR)-like ER kinase (PERK) (Figure 1; Perri et al., 2015). ATF6 is a member of the basic leucine zipper family of transcription factors (Wang et al., 2000). Under ER stress, ATF6 is translocated to the Golgi apparatus, where it is excised by site 1 and site 2 proteases to become an active transcription factor (Figure 1; Wang et al., 2000). Active ATF6 translocates to the nucleus and activates downstream target genes that function in protein folding and maturation, including glucose-regulated protein 78 (GRP78), glucose-regulated protein 94 (GRP94), and calreticulin (Schardt et al., 2010; Wang et al., 2016). ATF6 and XBP1 act to restore cell homeostasis and promote cell survival under ER stress (Yamamoto et al., 2004; Yoshida et al., 2006; Dadey et al., 2016).

PERK, a third branch of the UPR, functions to reduce the load of translated proteins that enter the ER and increases cell death under persistent ER stress (Lin et al., 2009; Kilberg et al., 2012). Following ER stress, PERK is oligomerized and activated by autophosphorylation (Harding et al., 2000). Active PERK phosphorylates eukaryotic translation initiation factor 2α (eIF2α) (Harding et al., 2000). Phosphorylated eIF2α inhibits eukaryotic translation initiation factor 2B (eIF2B) and decreases the assembly of the 43S initiation complex (Harding et al., 2000). This leads to translation arrest of most mRNAs while selectively allowing translation of specific proteins, such as activating transcription factor 4 (ATF4) (Harding et al., 2000). ATF4 regulates the expression of genes that function in amino acid metabolism and oxidative stress (Harding et al., 2003). Furthermore, ATF4 activates the transcription factor CCAAT/enhancer-binding protein homologous protein (CHOP), which plays a role in programmed cell death under persistent ER stress (Harding et al., 2000).

## Abnormalities in the UPR Pathway in BD

Several studies have reported a deficiency in UPR activation in lymphoblasts from BD patients (Kakiuchi et al., 2003; Kim et al., 2009; Pfaffenseller et al., 2014; Bengesser et al., 2016). Lymphoblast cells from BD patients have been used in many studies to characterize the pathology of BD, as access to live human brain tissue is not possible (Viswanath et al., 2015). An early study showed that the XBP1 single-nucleotide polymorphism (SNP) –116C→G is associated with an increased risk of developing BD (Kakiuchi et al., 2003). However, other studies failed to confirm the association of this SNP with BD (Hou et al., 2004; Kakiuchi et al., 2004). Additional work demonstrated that deficient UPR activation is caused by reduced transcription of the UPR target genes XBP1, GRP94, and CHOP in lymphoblast cells from BD patients after treatment with the ER stressors thapsigargin (TG) and tunicamycin (Tun) (So et al., 2007; Hayashi et al., 2009; Bengesser et al., 2018). This deficiency in UPR activation affects the ability to adapt to changes in the cellular environment, such as the accumulation of misfolded proteins in the ER, lipid overload, or changes in nutrient availability (Xu et al., 2005; Mandl et al., 2009). An inability of cells to adapt to ER stress results in increased cell death, and this has been demonstrated to occur in BD patient lymphocytes following treatment with Tun (Pfaffenseller et al., 2014). Increased neuronal cell death and neurodegeneration have been previously reported in BD patients (Karabulut et al., 2019; Gokcinar et al., 2020). Therefore, future studies should investigate whether ER stress and deficient UPR activation contribute to the neurodegeneration observed in BD patients.

## Mood Stabilizers Alter the Expression of UPR Target Genes

Studies have suggested modulation of the UPR pathway as a therapeutic target of mood stabilizers. VPA and Li increase the expression of the UPR chaperones GRP78, GRP94, and calreticulin in rat brain samples and cultured rat cells (Table 2). In this way, VPA has been shown to protect cells from different stress situations by inducing the UPR (Zhang et al., 2011; Li et al., 2017). For example, VPA protects SH-SY5Y cells from ER stress-induced apoptosis following treatment with TG by increasing the pro-survival protein GRP78 and reducing the pro-apoptotic protein CHOP (Li et al., 2017). Similarly, VPA protects cells from ischemia-reperfusion injuries in rats by attenuating the increase in CHOP levels (Zhang et al., 2011). However, certain studies have also demonstrated no effect on the UPR pathway by mood stabilizers. Although VPA increases GRP78 protein levels in HEK293 cells, neither VPA nor Li has a significant impact on the expression of GRP78 in Neuro-2a cells (Kakiuchi et al., 2003, 2009; Shi et al., 2007). Additionally, VPA and Li do not increase XBP1 expression in SH-SY5Y and lymphoblastoid cells (Kakiuchi et al., 2003). Nonetheless, there is strong support for UPR activation by mood stabilizers in the majority of mammalian studies conducted to date.

**FIGURE 2:**
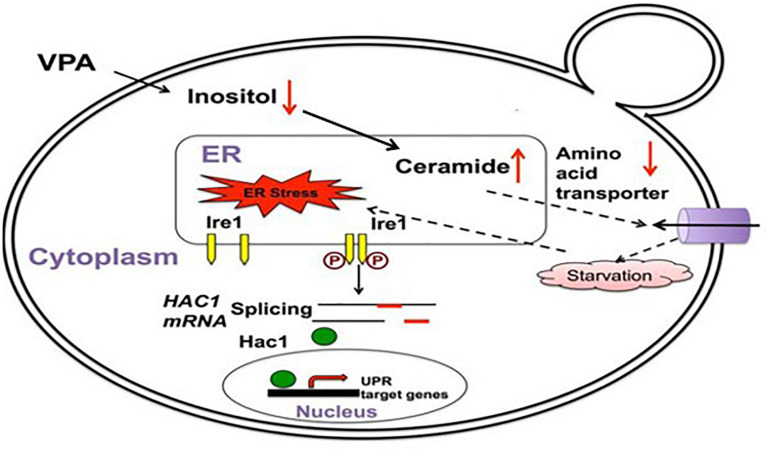
VPA induces the UPR pathway in yeast by increasing intracellular ceramide levels. VPA-mediated *myo*-inositol depletion results in elevated ceramide levels. Elevated ceramide results in the downregulation of amino acid transporters and subsequent UPR activation due to starvation stress. Figure adapted and modified from Jadhav et al. (2016).

Several mechanisms could explain how mood stabilizers activate the UPR. The first mechanism is through *myo*-inositol depletion and subsequent upregulation of ceramide levels. Abnormalities in *myo*-inositol levels have been observed in the brains of BD patients, and *myo*-inositol depletion has been hypothesized as part of the therapeutic mechanism of mood stabilizers (Shimon et al., 1997; Silverstone et al., 2005; Berridge, 2014). Studies have also reported alterations in the lipid profile, including changes in ceramide levels, in BD patients (Schwarz et al., 2008; Huang et al., 2018; Brunkhorst-Kanaan et al., 2019). An elegant study in yeast connected these observations and introduced a novel mechanism of UPR activation by the mood stabilizer VPA (Jadhav et al., 2016). Using yeast deficient in *myo*-inositol synthesis, Jadhav et al. (2016) demonstrated that depletion of intracellular *myo*-inositol by VPA upregulates ceramide levels and activates the UPR (Figure 2). In this study, it was shown that ceramide activates the UPR by inducing nutrient stress through the downregulation of plasma membrane amino acid transporters (Jadhav et al., 2016). It has also been shown that ceramide can activate the UPR pathway in human cells by inhibiting ER calcium uptake, suggesting that these ceramide-regulated mechanisms may work in tandem to induce the UPR following treatment with VPA (Liu et al., 2014). VPA-mediated activation of the UPR leads to increased expression of ER chaperones in yeast, including *KAR2*, the homolog of mammalian GRP78 (Jadhav et al., 2016). Upregulation of UPR chaperones indicates a protective effect of VPA. Therefore, *myo*-inositol depletion and UPR activation may be part of the same therapeutic mechanism employed by this drug. There is strong support for mood stabilizers leading to *myo*-inositol depletion in mammalian cells (Ye and Greenberg, 2015; Yu et al., 2017; Saiardi and Mudge, 2018). Additionally, it was shown that an increase in ceramide levels can activate the UPR pathway (Senkal et al., 2011; Liu et al., 2014). Future studies should characterize whether *myo*-inositol depletion activates the UPR in mammalian cells and whether this is dependent on an increase in ceramide levels.

A second mechanism is through inhibition of histone deacetylases (HDACs) (Shi et al., 2007). Support for this mechanism comes from a study showing that VPA, a known HDAC inhibitor, increases GRP78 expression, while VPA derivatives lacking the ability to inhibit HDACs do not increase GRP78 expression (Shi et al., 2007). In agreement with this, HDAC1 has been shown to bind to the promoter of GRP78 and repress its expression, suggesting that VPA may act at the transcriptional level to promote UPR activation by preventing repression by HDACs (Baumeister et al., 2009).

A third potential mechanism for UPR activation by mood stabilizers is through upregulation of the wolframin gene (WFS1) (Kakiuchi et al., 2009). WFS1 functions in mitigating ER stress, and WFS1 knockdown results in compensatory upregulation of GRP78, CHOP, and XBP1 in β-cells (Kakiuchi et al., 2009). In support of this mechanism, VPA has been shown to increase the expression of WFS1, leading to its dissociation from, and subsequent activation of, the ER chaperone GRP94 (Kakiuchi et al., 2009). Collectively, these studies support a protective role for the mood stabilizer VPA in the context of ER stress and highlight the possibility that VPA may act through more than one route to enhance the UPR response.

Regulation of the UPR pathway is linked to various aspects of brain function, and dysregulation is associated with the pathology of neurological disorders. GRP78 is an essential chaperone and a master regulator of the UPR, which functions in neuronal development (Weng et al., 2011). Abnormalities in GRP78 levels are associated with various neurological disorders such as Alzheimer’s and Parkinson’s diseases (Weng et al., 2011; Casas, 2017; Enogieru et al., 2019). Under normal conditions, GRP78 binds to the three UPR stress sensors, PERK, ATF6, and IRE1, and prevents their activation (Gong et al., 2017). However, under ER stress, GRP78 is released from these sensors by binding to unfolded proteins, resulting in sensor activation (Gong et al., 2017). Studies have suggested a role for the UPR in memory regulation, brain aging, neurotransmission, and in maintaining synaptic plasticity and structure in the central nervous system (Nosyreva and Kavalali, 2010; Freeman and Mallucci, 2016; Martínez et al., 2016; Miranda et al., 2020). Therefore, regulation of the UPR pathway may play a significant role in the pathophysiology and treatment of neurological disorders.

## Conclusion

The UPR pathway may play a significant role in the pathology and treatment of BD, a severe mood disorder that disrupts the lives of patients and their families (Gonda et al., 2012; Baldessarini et al., 2020). Li and VPA are two of the primary drugs used to treat BD patients (Geddes and Miklowitz, 2013). However, their efficacy is not universal, and they can cause serious side effects (Dreifuss et al., 1987; Pijl and Meinders, 1996; Mercke et al., 2000; Yonkers et al., 2004; Gitlin, 2016). The therapeutic mechanism of these drugs is unknown, which poses a challenge for developing better and more effective medications. Several studies have suggested that a deficiency in UPR activation is connected to BD pathology and that mood stabilizers may activate the UPR pathway as part of their therapeutic mechanisms (Tables 1, 2).

There are multiple mechanisms that could potentially underlie UPR activation by mood stabilizers, including HDAC inhibition, upregulation of WFS1, and *myo*-inositol depletion (Shi et al., 2007; Kakiuchi et al., 2009; Jadhav et al., 2016). While these mechanisms are not mutually exclusive, the *myo*-inositol depletion mechanism is currently the best supported for several reasons. Studies have rigorously demonstrated that BD drugs induce *myo*-inositol depletion in both yeast and mammalian cells, and *myo*-inositol is known to be especially essential for brain function, where the concentration can reach levels 20 times higher than in the blood (Vaden et al., 2001; Ye and Greenberg, 2015; Bizzarri et al., 2016; Jadhav et al., 2016; Yu et al., 2017; Saiardi and Mudge, 2018). To date, the most complete mechanistic study of UPR activation by mood stabilizers provides strong evidence for a causative link between VPA treatment, *myo*-inositol depletion, increased ceramide levels, and UPR activation in yeast (Figure 2) (Jadhav et al., 2016), and this is further corroborated by studies showing that *myo*-inositol and ceramide levels are aberrant in BD patients (Shimon et al., 1997; Silverstone et al., 2005; Schwarz et al., 2008; Brunkhorst-Kanaan et al., 2019). However, this mechanism has yet to be tested in mammalian cells, and it is within reason to speculate that HDAC inhibition and WFS1 upregulation could also contribute to the therapeutic mechanism of VPA.

Several limitations and discrepancies in the studies in Tables 1, 2 warrant further investigation. First, the small sample size in many of these studies decreases the statistical power of the results due to variations among individuals and populations (So et al., 2007). Thus, there is a need to characterize the UPR pathway in larger, more diverse populations of BD patients. Differences in the disease stage between patients may also contribute to this variability, with advanced stages reported to have a higher deficiency in UPR activation (Pfaffenseller et al., 2014). However, a challenge in resolving this issue is that BD is a psychiatric disorder with no unique biological marker that allows accurate characterization of the disease and its severity (Sigitova et al., 2017). Conducting large-scale genome-wide association (GWA) studies may aid in identifying biological markers for BD (Chuang and Kuo, 2017). The third limitation is that UPR deficiency has mainly been characterized in lymphoblast cells from BD patients despite the fact that BD is a neuronal disorder. To address this issue, postmortem studies should be used to investigate whether UPR activation is impaired in brain tissue from BD patients. Another fundamental challenge is that BD patients are often treated simultaneously with multiple drugs, making it challenging to separate UPR phenotypes associated with the disease itself vs. those resulting from mood stabilizers or other medications (Pfaffenseller et al., 2014). This matter could be addressed by characterizing the UPR pathway in BD patients who are not treated by mood stabilizers. However, a caveat to this is that recruiting untreated BD patients may pose both logistical problems (e.g., small sample sizes) and ethical dilemmas, as the disease is associated with high rates of suicide, especially in untreated patients (Gonda et al., 2012; Baldessarini et al., 2020). Therefore, the best strategy to clarify existing data is to focus research efforts on collecting and analyzing larger, more diverse data sets, identifying biological markers for BD, and utilizing postmortem studies to investigate UPR activation in the brain.

Uncovering the role of the UPR in the pathophysiology and treatment of BD would facilitate the development of more effective drugs to treat this debilitating and widespread disease. Additionally, to characterize the extent of UPR deficiency in BD patients, studies should investigate whether downstream targets of UPR activation are also impaired in BD patients. For example, a deficiency in UPR activation may lead to abnormalities in lipid metabolism, mitochondrial function, protein secretion, or calcium signaling (Gong et al., 2017). Therefore, drugs could be designed to target specific signaling branches of the UPR pathway or act to resolve the downstream deficiencies of UPR activation. These drugs could be chemical chaperones such as 4-Phenylbutyric acid (PBA), which alleviates ER stress by assisting protein folding in the ER, and is already approved by the FDA to treat urea cycle disorder (Roy et al., 2015). Ultimately, specific and effective drugs with fewer side effects will reduce the severity of this disease and improve the lives of BD patients.

## Author Contributions

MSu, MSc, and MG wrote sections of the manuscript. All authors contributed to manuscript revision, read, and approved the submitted version.

## Conflict of Interest

The authors declare that the research was conducted in the absence of any commercial or financial relationships that could be construed as a potential conflict of interest.

## Publisher’s Note

All claims expressed in this article are solely those of the authors and do not necessarily represent those of their affiliated organizations, or those of the publisher, the editors and the reviewers. Any product that may be evaluated in this article, or claim that may be made by its manufacturer, is not guaranteed or endorsed by the publisher.
